# 535 Influence of Covid-19 Pandemic Conditions on the Features of Pediatric Minor Burn Injuries

**DOI:** 10.1093/jbcr/irad045.132

**Published:** 2023-05-15

**Authors:** Ayse Ebru Abali, Cem Aydogan, Semra Kamilova, Nigar Turkmen, Santiago Santelis, Mehmet Haberal

**Affiliations:** Baskent University, Ankara, Ankara; Baskent University, Ankara, Ankara; Başkent University, Ankara, Ankara; Başkent University, Ankara, Ankara; Burn Center and Burn and Fire Disasters Institute, Baskent University, Sanford, Florida; Baskent University, Ankara, Ankara

## Abstract

**Introduction:**

Majority of pediatric burns are minor injuries. Social life changes due to Covid-19 pandemic may have influenced their characteristics. We compared the features of pediatric burn cases which were treated as outpatients at our center before and during Covid-19 pandemic.

**Methods:**

The records of 658 patients were reviewed. The study group was evaluated in two groups; Group I: patients-treated between March 2018 and February 2020 (pre-pandemic) (n=350), Group II: patients-treated between March 2020 and February 2022(Pandemic) (n=308). Data collected for each case were age; sex; insurance status, place of residence; burn-causes, extent of burns; body-site affected; time and environment where the injury occurred, time-interval between occurrence of injury and admissions to the burn-center; (mean± SE, p<.05).

**Results:**

Mean age (years) was 4,32±0,24 for Group I, it was 4,32±0,18 for Group II; male:female ratio was 0,87:1 in Group I, it was 1,1: 1 in Group II; mean total surface area burned was 1,94±0,14 % (min:0,5, max:16) in Group I, and it was 1,98±0,26% (min:0,1 max: 16,1) in Group II (p>.05). Most of the patients were from urban areas and were covered by social security system in both groups (p>.05). Injuries occurred between 06:00 and 18:00 in 63,7% of Group I and in 62,6% of Group II (p>.05). The most common scenario was the domestic environment for both groups with a rise in rates of outdoor-burns Group II (p<.05) and this rise was observed during the ‘new normal’ period, but not during the ‘lock-down’(p< .05). The most common burn-cause was the scalds for both groups (p >.05). Hands, trunk, head and neck regions were less frequently involved in group II (p<.05). Rates of direct admission were similar for both, but children in Group II were more frequently brought to our burn center on the day of injury (p<.05).

**Conclusions:**

The decrease in injuries to hands, trunk, head and neck, and the increase in admissions on the day of injury were remarkable during pandemic period. These results may be clues for enhanced care-giver precautions against injuries during ‘stay home’ days. Increased frequency of outdoor burns during ‘new normal’ may indicate the negative effects of this new unfamiliar social life on care-giver watchfulness. However, increased admissions on the day of the injury may be influenced both by the changing social conditions of care-givers and the uninterrupted service of our burn center while others were serving for covid-19 patients rather than burn victims.

**Applicability of Research to Practice:**

This study helped to make recommendations for evidence-based interventions to decrease factors associated with pediatric minor burn injuries before, during and after pandemic

